# Tiotropium Is Predicted to Be a Promising Drug for COVID-19 Through Transcriptome-Based Comprehensive Molecular Pathway Analysis

**DOI:** 10.3390/v12070776

**Published:** 2020-07-20

**Authors:** Keunsoo Kang, Hoo Hyun Kim, Yoonjung Choi

**Affiliations:** 1 Department of Microbiology, College of Science & Technology, Dankook University, Cheonan 31116, Korea; rlagngus@gmail.com; 2 Deargen Inc., Daejeon, Yuseong-gu, Munji-dong 103-6, Korea

**Keywords:** SARS-CoV-2, COVID-19, tiotropium, molecular pathways, RNA-seq, COPD

## Abstract

The coronavirus disease 2019 (COVID-19) outbreak caused by severe acute respiratory syndrome coronavirus 2 (SARS-CoV-2) affects almost everyone in the world in many ways. We previously predicted antivirals (atazanavir, remdesivir and lopinavir/ritonavir) and non-antiviral drugs (tiotropium and rapamycin) that may inhibit the replication complex of SARS-CoV-2 using our molecular transformer–drug target interaction (MT–DTI) deep-learning-based drug–target affinity prediction model. In this study, we dissected molecular pathways upregulated in SARS-CoV-2-infected normal human bronchial epithelial (NHBE) cells by analyzing an RNA-seq data set with various bioinformatics approaches, such as gene ontology, protein–protein interaction-based network and gene set enrichment analyses. The results indicated that the SARS-CoV-2 infection strongly activates TNF and NFκB-signaling pathways through significant upregulation of the *TNF*, *IL1B*, *IL6*, *IL8*, *NFKB1*, *NFKB2* and *RELB* genes. In addition to these pathways, lung fibrosis, keratinization/cornification, rheumatoid arthritis, and negative regulation of interferon-gamma production pathways were also significantly upregulated. We observed that these pathologic features of SARS-CoV-2 are similar to those observed in patients with chronic obstructive pulmonary disease (COPD). Intriguingly, tiotropium, as predicted by MT–DTI, is currently used as a therapeutic intervention in COPD patients. Treatment with tiotropium has been shown to improve pulmonary function by alleviating airway inflammation. Accordingly, a literature search summarized that tiotropium reduced expressions of *IL1B*, *IL6*, *IL8*, *RELA, NFKB1* and TNF in vitro or in vivo, and many of them have been known to be deregulated in COPD patients. These results suggest that COVID-19 is similar to an acute mode of COPD caused by the SARS-CoV-2 infection, and therefore tiotropium may be effective for COVID-19 patients.

## 1. Introduction

The coronavirus disease (COVID-19) outbreak caused by the severe acute respiratory syndrome coronavirus 2 (SARS-CoV-2) [1] has become a pandemic like the 1918 influenza pandemic [2], which was the most severe pandemic in recent history. The molecular mechanisms of extreme contagious nature and acute severity of SARS-CoV-2 are still unknown. However, some hypotheses about the potential pathogenesis of COVID-19 suggest that immunological changes caused by the SARS-CoV-2 infection proceed with the onset of pneumonia and respiratory failure [3]. Indeed, patients die mostly from severe multiple organ dysfunction, acute respiratory distress syndrome (ARDS), heart failure and renal failure, with uncontrolled immunological signatures [4]. These uncontrolled and overexpressed proinflammatory genes may lead to a phenomenon called cytokine storm [5] which has been proposed to be diagnosed in a severe COVID-19 patient subgroup.

SARS-CoV-2 manipulates host defense systems, leading to overexpression of proinflammatory cytokines such as interleukin 6 (*IL-6*), interleukin 1B (*IL-1B*) and tumor necrosis factor (*TNF*) [6]. The deregulation of host immune systems is thought to eventually induce fatal damage to multiple organs. Similarly, elevated levels of cytokine gene expressions including C–C motif chemokine ligand 2 (*CCL2*, also known as monocyte chemoattractant protein 1 [*MCP-1*]), C–X–C motif chemokine ligand 10 (*CXCL10*), C–X–C motif chemokine ligand 9 (*CXCL9*) and C–X–C motif chemokine ligand 8 (*CXCL8*, also known as *IL-8*) were also observed in H5N1 avian influenza virus-infected patients with severe lung injury [7]. In particular, IL-1B participates as a key player in inflammatory response during early infections [8]. In addition, IL-6 and IL-8 are associated with overall inflammation, including fever, immune cell recruitment, immune cell stimulation, vasodilation and other inflammatory responses [8]. Upregulation of such proinflammatory genes supports pneumonic pathogenesis of COVID-19. Therefore, mitigating such a cytokine storm by immunosuppressant drugs may help a subgroup of COVID-19 patients with severe acute respiratory failure.

An important aspect for understanding molecular mechanisms underlying the SARS-CoV-2 infection is the process of entering the host cells. SARS-CoV-2, which is similar to severe acute respiratory syndrome coronavirus (SARS-CoV), requires the angiotensin-converting enzyme 2 (ACE2) as a receptor to enter the host cells [1]. The ACE2 receptor is abundantly expressed in human airway epithelia and protects the lungs from acute lung failure [9]. The interaction between the spike protein of SARS-CoV-2 and ACE2 decreases the availability of ACE2, which results in lung injury [10]. Interestingly, regulation of renin–angiotensin II systems by ACE2 is also crucial for the pathogenesis of chronic obstructive pulmonary diseases (COPD) [11]. Although there is no apparent evidence that the treatment of asthma or COPD may have contributed to prevent COVID-19, it has recently been reported that the prevalence of asthma and COPD in patients diagnosed with COVID-19 is much lower than that in the general population [12]. In addition, a recent study showed that inhaled corticosteroids, which are treatment options for COPD patients, downregulate the SARS-CoV-2 receptor ACE2 in COPD through suppression of type I interferon [13].

Chronic obstructive pulmonary disease is characterized by respiratory symptoms such as dyspnea, cough, sputum production and airflow limitation [14]. For the treatment of COPD, tiotropium is a prevalent drug that can provide a significant improvement of the lung function [15,16,17,18]. Tiotropium is a long-acting, antimuscarinic bronchodilator that can produce the smooth muscle relaxation and bronchodilation and acts on the airway smooth muscle through M3 muscarinic receptors [17,18,19]. It can attenuate the dyspnea and relieve the muscle contraction in patients. Interestingly, tiotropium can be also used to control asthma. Asthma is a chronic inflammatory disease of the respiratory system and causes severe symptoms of airway inflammation, such as cough, shortness of breath, chest tightness and wheezing [20,21].

Given that tiotropium can apply to multiple respiratory diseases, we hypothesize that it may have some impact on COVID-19. One small piece of evidence for our hypothesis comes from our previous study that identified several antiviral and non-antiviral drugs for COVID-19 [22]. Briefly, we previously proposed that several antiviral drugs, such as atazanavir, remdesivir and Kaletra (lopinavir/ritonavir), may be effective in inhibiting SARS-CoV-2 and it was based on the affinity prediction using our MT–DTI deep-learning-based drug–target interaction algorithm [22,23]. In the analysis, non-antiviral drugs, including rapamycin (immunosuppressor) and tiotropium bromide, were also identified as promising candidates for SARS-CoV-2. However, there was no evidence supporting the prediction. We questioned why the MT–DTI artificial intelligence model predicted tiotropium, a long-acting, antimuscarinic bronchodilator used in maintaining COPD and asthma, would be a promising drug that may act on the replication complex of SARS-CoV-2.

The purpose of this study is to define the molecular pathology of COVID-19 and to find evidence of top-ranked repurposed-drug candidates identified by our MT–DTI deep-learning-based drug–target affinity prediction model. To this end, we reanalyzed a recently available transcriptome data set, which was performed in normal human bronchial epithelial (NHBE) cells infected with SARS-CoV-2 [24]. We used various bioinformatic analyses to define molecular pathogenesis of COVID-19 and found that the predicted molecular mechanism of SARS-CoV-2’s pathogenicity was similar to the progression of COPD (or asthma). Based on the molecular pathogenesis of COVID-19 defined in this study and its similarity to COPD, the association between COVID-19 and COPD should be further investigated in the near future.

## 2. Materials and Methods

### 2.1. RNA-seq Data Analysis

A previous study [24] profiled transcriptomes in normal human bronchial epithelial cells infected with mock (control) or SARS-CoV-2 (USA-WA1/2020) as well as A549 lung cancer cells infected with mock (control), SARS-CoV-2, influenza A virus (IAV; Puerto Rico/8/1934; H1N1) or respiratory syncytial virus (RSV). The read count table, which contains the number of mapped reads in all samples at the gene level, was downloaded from the gene expression omnibus (GEO) data repository (https://www.ncbi.nlm.nih.gov/geo/query/acc.cgi?acc=GSE147507, GSE147507). A batch effect correction between the groups was conducted using RUVSeq [25]. Differentially expressed genes (DEGs) were identified using DESeq2 [26] with an adjusted *p*-value cutoff of 0.05. DEGs showing less than 1.5-fold-change between conditions were further discarded.

### 2.2. Network Analysis

A list of upregulated DEGs (Table S1) was used to construct lung tissue-specific protein–protein interaction (PPI) networks using NetworkAnalyst (https://www.networkanalyst.ca/) with the minimum-order or zero-order network models [27]. For this analysis, 171 upregulated genes were used as an input for NetworkAnalyst. Then, the lung tissue-specific PPI network option was selected with the default filter parameter of 15.0. The zero-order (or minimum) network was used to construct a PPI-based network.

### 2.3. Gene Set Enrichment Analysis

Gene set enrichment analysis (GSEA) was conducted using the fast gene set enrichment analysis (FGSEA) method [28]. Hallmark (H), positional (C1), curated (C2), regulatory target (C3), GO (C5), oncogenic (C6) and immunologic (C7) gene sets from the molecular signatures database (version 7.1, https://www.gsea-msigdb.org/gsea/msigdb/index.jsp) were used for the analysis.

### 2.4. Transcription Factor Binding Motif Prediction

Transcription factor binding motifs in the promoter regions of upregulated genes were predicted using PSCAN (http://159.149.160.88/pscan/) [29]. The promoter region was set to 500 bp (−450 bp ~TSS ~+50 bp) around transcription start sites (TSSs) of the genes and the Jaspar (2018_NR) motif data set was used to search for motifs in the promoter regions.

### 2.5. Gene Ontology Analysis

Upregulated DEGs were annotated and functionally classified using the PANTHER gene ontology (GO) analysis application (http://pantherdb.org/) [30]. In addition, the same DEGs were also analyzed using g:Profiler (https://biit.cs.ut.ee/gprofiler/gost) with default parameters [31]. The parameters were set as follows: ‘only annotated genes’ for the statistical domain scope, ‘g:SCS threshold’ for the significance threshold and 0.05 for the user threshold.

### 2.6. Visualization

Morpheus (https://software.broadinstitute.org/morpheus/) was used to draw heatmaps showing expression levels of given genes. The expression levels of the genes were normalized using the min–max normalization method [32]. The genes were clustered using the Hierarchical clustering algorithm with the average linkage method [33]. The one-minus Pearson correlation metric was used for clustering [34].

## 3. Results

### 3.1. Signaling Pathways Upregulated by SARS-CoV-2 Infection in Normal Human Bronchial Epithelial Cells

**Figure 1:**
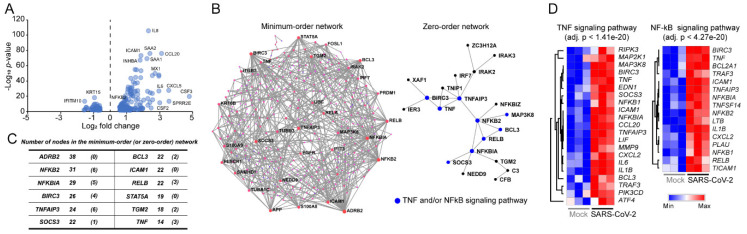
Analysis of differentially expressed genes in severe acute respiratory syndrome coronavirus 2 (SARS-CoV-2)-infected normal human bronchial epithelial (NHBE) cells compared to mock-infected cells. (**A**) Volcano plot shows that up- and downregulated differentially expressed genes (DEGs) in SARS-CoV-2-infected NHBE cells compared to mock-infected control cells; (**B**) Minimum-order and zero-order protein–protein interaction (PPI) networks were constructed with 171 upregulated DEGs using NetworkAnalyst (https://www.networkanalyst.ca/); (**C**) Genes containing higher number of PPI interactions are listed; (**D**) Most significantly upregulated pathways identified in the PPI networks are shown. Heatmaps were generated using Morpheus (https://software.broadinstitute.org/morpheus/) after the hierarchical clustering analysis with the average-linkage method.

To gain molecular insights on how the SARS-CoV-2 infection interferes with molecular signaling pathways in host cells, we reanalyzed RNA-seq data sets (GSE147507) performed in SARS-CoV-2-infected normal human bronchial epithelial (NHBE) cells cultured in bronchial epithelial growth media supplemented with BEGM SingleQuots [24]. After batch effect removal using RUVSeq [25], differentially expressed genes (DEGs) were identified between mock-infected NHBE (control) and SARS-CoV-2-infected NHBE cells using DESeq2 [26]. Totals of 171 up- and 56 downregulated DEGs were identified with an adjusted p-value cutoff of 0.05 (Figure 1A and Table S1). As expected, many of the upregulated genes were classified into cytokine-related gene sets such as granulocyte-macrophage colony-stimulating factor (*CSF2*, also known as GM-CSF), interleukin 8 (*IL-8*, also known as *CXCL8*) and interleukin 6 (*IL-6*). Particularly, the tumor necrosis factor (*TNF*), intercellular adhesion molecule 1 (*ICAM1*) and nuclear factor kappa B subunit 2 (*NFKB2*) genes, which belong to the TNF- and/or NFκB-signaling pathways, were significantly upregulated. To gain further biologic insights, taking into account protein–protein interactions (PPI), we constructed lung tissue-specific PPI networks using NetworkAnalyst [27] with the identified upregulated DEGs (*n* = 171). The results showed that ADRB2, NFKB2, NFKBIA, TNFAIP3, RELB, TNF and ICAM1 were the most highly connected proteins that may regulate various signaling pathways through PPI (Figure 1B). Interestingly, the beta-2-adrenergic receptor (*ARDB2*) gene, which is abundantly expressed in bronchial smooth muscle cells and plays important roles in regulating various systems, including cardiac, pulmonary and vascular systems [35], was one of mostly connected protein in the minimum-order network (Figure 1C). NFKB2 and TNFAIP3 were the proteins showing the highest PPI interactions in the zero-order network. Gene ontology analysis of the components of the constructed minimum-order PPI network also indicated that genes involved in the TNF- (adjusted *p*-value < 1.41e^−20^) and/or NFκB-signaling (adjusted *p*-value < 4.27e^−20^) pathways were significantly overrepresented in the upregulated DEGs (Figure 1D). Overall, the DEG and PPI network analyses indicated that the *NFKB2*, mitogen-activated protein kinase kinase kinase 8 (*MAP3K8*), BCL3 transcription coactivator (*BCL3*), RELB proto-oncogene (*RELB*), NFKB inhibitor alpha (*NFKBIA*), suppressor of cytokine-signaling 3 (*SOCS3*), TNF alpha induced protein 3 (*TNFAIP3*), baculoviral IAP repeat containing 3 (*BIRC3*), *TNF _and_ ICAM1* genes, which were significantly upregulated by SARS-CoV-2 infection, exert a profound influence on NHBE cells through the TNF- and/or NFκB-signaling pathways.

### 3.2. Decoding Upregulated Signaling Pathways Caused by SARS-CoV-2

**Figure 2:**
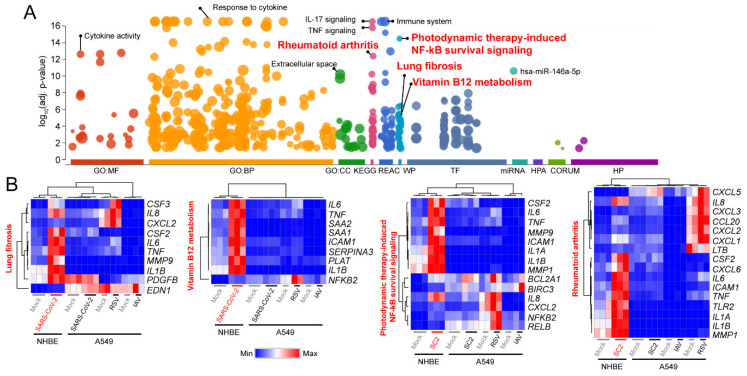
Gene ontology analysis of the upregulated differentially expressed genes (DEGs). (**A**) g:Profiler (https://biit.cs.ut.ee/gprofiler/) was used to dissect molecular pathways of 171 upregulated genes in SARS-CoV-2-infected NHBE cells compared to mock-infected control cells with the following categories: GO:MF—molecular function; GO:CC—cellular component; GO:BP—biologic process; KEGG—Kyoto Encyclopedia of Genes and Genomes; REAC—reactome; WP—Wikipathways; TF—TRANSFAC; MIRNA—miRTarBase; HPA—human protein atlas; CORUM—CORUM protein complexes; HP—human phenotype ontology. (**B**) Heatmaps of lung-fibrosis pathway, vitamin B12 metabolism, photodynamic therapy-induced NFκB survival-signaling, and rheumatoid arthritis pathways are shown. SC2—SARS-CoV-2.

The TNF- and/or NFκB-signaling pathways are well known signaling pathways that are involved in host immune response [36]. Therefore, if they are not tightly controlled as a defense mechanism for pathogens, massive uncontrolled proinflammatory cytokine production called cytokine storm can be raised. It ultimately leads to severe complications or death as seen in some fatal cases of H5N1 influenza infection [7]. However, the exact mechanisms that contribute to the cytokine storm are unclear. To decode signaling pathways underlying the molecular pathogenesis of COVID-19 deeply, we conducted a comprehensive gene ontology analysis with 171 upregulated DEGs in SARS-CoV-2-infected NHBE cells compared to corresponding control samples using g:Profiler [31]. As expected, the results showed that many of the genes involved in cytokine-signaling, such as IL-17 (adjusted *p*-value < 2.90e^−21^) and TNF (adjusted *p*-value < 1.74e^−16^) signaling pathways, were significantly upregulated (Figure 2A and Table S2). Interestingly, we also found that some disease-related pathways, such as ‘lung fibrosis’ (adjusted *p*-value < 3.70e^−06^) and ‘rheumatoid arthritis’ (adjusted *p*-value < 4.36e^−13^), were significantly associated with the upregulated DEGs. Most of these genes were shared among the pathways. For example, the *IL8*, C–X–C motif chemokine ligand 2 (*CXCL2*), *CSF2*, *IL6*, *TNF* and interleukin 1 beta (*IL1B*) genes were overlapped between the ‘lung fibrosis’ and ‘rheumatoid arthritis’ pathways, while the *IL6*, *TNF*, *ICAM1*, *IL1B*, and *NFKB2* genes were common in the ‘vitamin B12 metabolism’ and ‘photodynamic therapy-induced NF-kB survival-signaling’ pathways (Figure 2B). In addition, most of the identified genes in these pathways were upregulated in SARS-CoV-2-infected NHBE cells or RSV-infected A549 cells, but not in SARS-CoV-2-infected A549 cells (Figure 2B). Collectively, these results showed that the SARS-CoV-2 infection in the NHBE cells activated host defense pathways (cytokine and immune system) as well as the pathways (lung fibrosis and rheumatoid arthritis) that have negative effects on the host cells.

### 3.3. Comparison of Transcriptomic Changes Caused by SARS-CoV-2, Respiratory Syncytial Virus and Influenza A Virus

**Figure 3:**
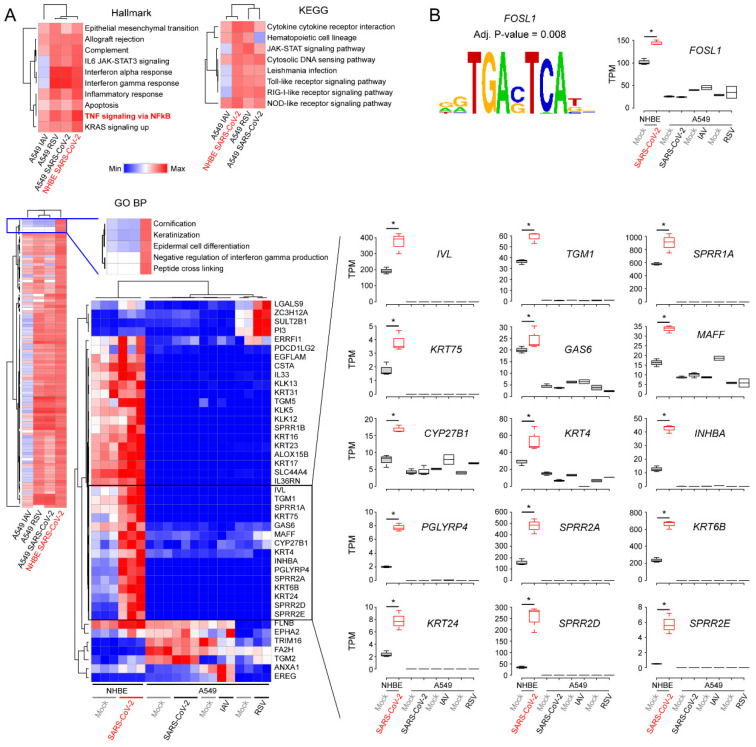
Gene set enrichment analysis of expressed genes. (**A**) Gene set enrichment analysis was conducted using FGSEA [28] with expressed gene sets (the base mean value of DESeq2 > 10). Ranking of genes in a gene set was defined by the fold-change value. Heatmaps (top) were constructed with normalized enrichment score (NES). The heatmap (bottom) shows min–max normalized expression levels of genes in the GO:BP category. Boxplots show expression levels of given genes in each sample. Hallmark—hallmark gene sets; KEGG—Kyoto Encyclopedia of Genes and Genomes; GO:BP—biologic process; TPM—transcripts per million; (**B**) Transcription factor binding motifs overrepresented in the promoter regions of the genes (black–box in the heatmap) was identified using PSCAN (http://159.149.160.88/pscan/). *P-*values were calculated using Student’s t-test (*p-value: < 0.01).

As we found some differently upregulated pathways between SARS-CoV-2-infected NHBE cells and SARS-CoV-2-infected, RSV-infected and IAV-infected A549 cells, we performed a massive gene set enrichment analysis to identify unique gene sets that could explain the acute severity shown in the COVID-19 patients [37]. More than 9000 gene sets covering various defined categories from the molecular signatures database (https://www.gsea-msigdb.org/gsea/msigdb/index.jsp) were analyzed. We used the following 7 different gene sets for the analysis: ‘hallmark gene sets’, ‘positional gene sets’, ‘curated gene sets’, ‘regulatory target gene sets’, ‘GO gene sets’, ‘oncogenic gene sets’ and ‘immunologic gene sets’. The analysis revealed that there were common and unique gene sets in response to the virus infection. For example, ‘IL6 JAK-STAT3-signaling’, ‘interferon alpha response’ and ‘interferon gamma response’ pathways were upregulated in IAV-infected A549, SARS-CoV-2-infected A549 and SARS-CoV-2-infected NHBE cells compared to corresponding mock-infected cells, whereas they were downregulated in IAV-infected A549 cells according to the Hallmark database (Figure 3A and Table S3). In contrast, ‘TNF-signaling via NFκB’, ‘inflammatory response’, and ‘complement’ were the upregulated pathways common in all the virus-infected cells. Similar patterns were also observed according to the KEGG database (Figure 3A). Next, we focused on the unique molecular pathway observed in the SARS-CoV-2-infected NHBE cells because A549 cells are less likely to be infected by SARS-CoV-2 due to low expression of the viral receptor ACE2 [38]. Among 7530 known pathways in the gene ontology biologic pathway (GO BP) database, the ‘cornification’, ‘keratinization’, ‘epidermal cell differentiation’, ‘negative regulation of interferon gamma production’ and ‘peptide cross linking’ pathways unique to the SARS-CoV-2-infected NHBE cells were identified (Figure 3A). Since most of the genes between these pathways were shared, we constructed a heatmap to pinpoint which genes were mostly changed in SARS-CoV-2-infected NHBE cells. The result indicated that involucrin (*IVL*), transglutaminase 1 (*TGM1*), small proline rich protein 1A (*SPRR1A*), keratin 75 (*KRT75*), growth arrest specific 6 (*GAS6*), MAF BZIP transcription factor F (*MAFF*), cytochrome P450 family 27 subfamily B member 1 (*CYP27B1*), keratin 4 (*KRT4*), inhibin subunit beta A (*INHBA*), peptidoglycan recognition protein 4 (*PGLYRP4*), small proline rich protein 2A (*SPRR2A*), keratin 6B (*KRT6B*), keratin 24 (*KRT24*), small proline rich protein 2D (*SPRR2D*), and small proline rich protein 2E (*SPRR2E*) genes were the most highly upregulated genes among these pathways (Figure 3A). In addition, the kallikrein family of serine proteases (*KLK13*, *KLK5* and *KLK12*) and keratin (*KRT16*, *KRT17*, *KRT23* and *KRT31*) and small proline rich protein 1B (*SPRR1B*) genes were also upregulated uniquely to the SARS-CoV-2-infected NHBE cells. To understand the molecular mechanism, potentially controlling these genes, we searched for an upstream transcription factor by scanning promoter sequences of the genes. Intriguingly, the FOSL1 (also known as fos-related antigen 1 or FRA1) binding motif was significantly overrepresented (adjusted *p*-value < 0.008) (Figure 3B). Concordantly, the expression level of the *FOSL1* gene was also significantly upregulated in SARS-CoV-2-infected NHBE cells compared to mock-infected NHBE cells. Overall, these results showed that SARS-CoV-2 infection in NHBE cells altered various pathways of the host cells and the genes associated with the keratinization, cornification, epidermal cell differentiation and peptide cross linking were uniquely upregulated.

### 3.4. Functional Classification of SARS-CoV-2-Activating Genes

**Figure 4:**
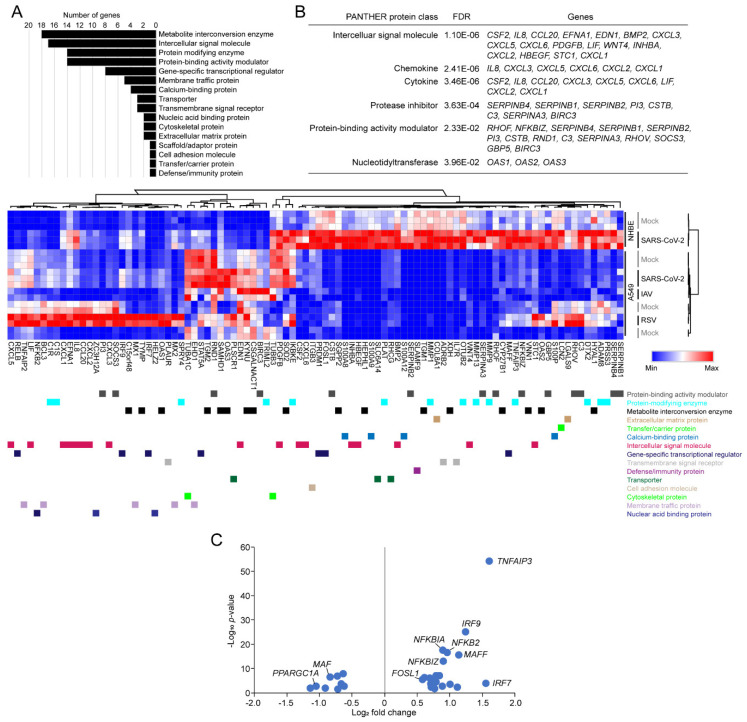
Functional classification of the upregulated genes. (**A**) Protein class-based gene ontology analysis was conducted using PANTHER (http://pantherdb.org/) with 171 upregulated DEGs; (**B**) Statistical overrepresentation test of the 171 upregulated DEGs with the PANTHER protein-class database was conducted with Fisher’s exact test (top). Heatmap (bottom) shows min–max-normalized expression levels of genes in the PANTHER protein-class database. FDR—false discover rate-corrected *p*-value; (**C**) The volcano plot shows that up- and downregulated differentially expressed genes, encoding transcription factors in SARS-CoV-2-infected NHBE cells compared to mock-infected control cells.

When a cell is infected by viruses, many signaling pathways are activated through designated signaling cascades. These are mediated by complex processes, and therefore it is difficult to clearly identify which signaling pathway is beneficial to the host or, conversely, to the virus. Nevertheless, we classified upregulated genes in SARS-CoV-2-infected NHBE cells into known protein classes using PANTHER [30]. We have provided the list of classified genes in Table S4. As expected, many upregulated genes were belonging to the metabolite interconversion enzyme, intercellular signal molecule, protein modifying enzyme, protein-binding activity modulator and gene-specific transcriptional regulatory pathways (Figure 4A). Similar to the NetworkAnalyst (Figure 1), g:Profiler (Figure 2) and FGSEA (Figure 3) gene ontology analysis applications, PANTHER also identified similar pathways in SARS-CoV-2-infected NHBE cells. Among those, the genes encoding protease inhibitors (false discovery rate-adjusted *p*-value < 3.63e^−04^) such as serpin family B member 4 (*SERPINB4*), serpin family B member 1 (*SERPINB1*), serpin family B member 2 (*SERPINB2*), peptidase inhibitor 3 (*PI3*), cystatin B (*CSTB*), complement C3 (*C3*), serpin family A member 3 (*SERPINA3*), and *BIRC3* were newly identified (Figure 4B). Hierarchical clustering of annotated upregulated genes revealed that approximately a half of the annotated upregulated genes were relatively more expressed in SARS-CoV-2-infected NHBE cells than those of SARS-CoV-2-, RSV- and IAV-infected A549 cells. However, the difference may be due to the apparent cell-type difference between NHBE and A549 cells. Nevertheless, the results clearly indicated that there would be strong induction of genes associated with anti- or pro-viral activities when normal bronchial epithelial cells were infected by SARS-CoV-2. This uncontrolled upregulation of genes may be the cause of acute complications, such as acute respiratory distress syndrome (ARDS), shown in some of COVID-19 patients through pulmonary fibrosis and/or cytokine storms [6,39]. To understand molecular mechanisms underlying these phenomena, we investigated changes in expression levels of transcription factors. Intriguingly, we found that *TNFAIP3*, interferon regulatory factor 9 (*IRF9*), *NFKBIA*, *NFKB2*, NFKB inhibitor zeta (*NFKBIZ*), MAF bZIP transcription factor F (*MAFF*) and interferon regulatory factor 7 (*IRF7*) were significantly upregulated, whereas MAF bZIP transcription factor (*MAF*) and PPARG coactivator 1 alpha (*PPARGC1A*) were downregulated in SARS-CoV-2-infected NHBE cells (Figure 4C). Additional experimental and clinical evidence should be supported to interpret this predicted molecular pathogenesis of COVID-19 and ultimately, to develop targeted therapeutics.

### 3.5. Tiotropium as a Promising Drug Candidate for COVID-19

There is an urgent need for finding effective treatment for COVID-19 patients. We had ranked available drugs based on affinity prediction using our MT–DTI deep-learning-based model and proposed available antiviral drugs that may act on inhibiting the replication complex of SARS-CoV-2 in a previous study [23]. However, the previous analysis indicated that several non-antiviral drugs also seemed to be potent candidates, showing higher binding affinity scores than antivirals. Nevertheless, we failed to show any evidence for those drugs in the previous study [23]. As a follow-up study, we surveyed whether some of the top-ranked non-antiviral drugs predicted by the MT–DTI model may reduce the symptoms of COVID-19, based on the molecular pathogenesis dissected in this study. To this end, we searched for experimental evidence through the literature to address the antiviral effects of the top two drugs, rapamycin (Sirolimus) and tiotropium. We found that these drugs have an inhibitory effect on some of the SARS-CoV-2-induced genes identified in this study. The results are summarized in Table 1. According to the survey, we propose that tiotropium is one of the most promising drugs that can reduce symptoms of COVID-19 for the following reasons. First, the predicted binding affinity of tiotropium against SARS-CoV-2’s replication complex was the best out of an FDA-approved drug library in our previous study [23]. It means that tiotropium may directly bind to some components of the replication complex and this most likely leads to the inhibition of SARS-CoV-2. Interestingly, tiotropium showed some antiviral effects on rhinovirus-infected human airway epithelial cells [40], RSV-infected human epithelial type 2 (HEp-2) cells [41] and lung tissues from cigarette smoke-exposed and RSV-infected mice [42]. Second, tiotropium has been shown to inhibit the TNF- and/or NFκB-signaling pathways, which were the most significantly upregulated pathways upon the infection of SARS-CoV-2 in NHBE cells. Specifically, several studies demonstrated that tiotropium reduced expression levels of *NFKB1*, *RELA*, *ICAM1*, *IL-1B*, *IL-6*, *IL-8*, matrix metallopeptidase 1 (*MMP1*) or *TNF* (Table 1). Consequently, it suppressed inflammation in humans, mice, rats and cats. Tiotropium also attenuated virus-induced pulmonary inflammation in cigarette smoke-exposed mice through the reduction of *IL-6*, *CXCL1* (previously called KC) and *TNF* in the lung [42]. On the other hand, another potent drug rapamycin, which is an immunosuppressant inhibiting the mammalian target of rapamycin (mTOR) pathway, showed some unfavored effects such as augmenting LPS-induced lung injury [43,44], while it also reduced inflammation by inhibiting the NFκB-signaling pathway through the mTOR pathway. Lastly, as a clinically proven drug for chronic obstructive pulmonary disease (COPD) [16] and asthma [45], there are several lines of evidence showing that tiotropium improved lung functions [46]. Therefore, we recommend that it is worthwhile to test whether tiotropium can alleviate the symptoms of COVID-19 and prevent disease progression.

## 4. Discussion

In this study, we attempted to define the molecular pathogenesis of COVID-19 by reanalyzing a transcriptome data set of SARS-CoV-2-infected NHBE cells [24] using various bioinformatic approaches. The previous study from which the RNA-seq data sets were originated had compared transcriptomes of NHBE cells infected by SARS-CoV-2 with A549 cells infected by SARS-CoV-2, respiratory syncytial virus (RSV) or influenza A virus (IAV) [24]. However, the analysis was not comprehensive so that further biologic insights could be obtained. To this end, we conducted a comprehensive pathway analysis by dissecting transcriptomes into known pathways using four different gene set analysis tools, NetworkAnalyst, g:Profiler, FGSEA and PANTHER. Our analysis discovered the following biologic insights to consider in order to develop effective treatment options for COVID-19. First, the SARS-CoV-2 infection in normal bronchial epithelial cells resulted in upregulation of the genes involved in the TNF- and/or NFκB-signaling pathways. This activation may lead to favorable or unfavorable gene expression changes for host cells and contribute to ARDS shown in some COVID-19 patients [56]. In addition to the pathways, inflammatory chemokines, such as C–X–C motif chemokine ligand 1 (*CXCL1*), *CXCL2*, *CXCL3*, *CXCL5* and *CXCL6*, were significantly upregulated as previously reported [24], while *CXCL14* with anti-inflammatory activity [57] was substantially downregulated (Table S1). Several interleukin genes, including interleukin 1 alpha (*IL1A*), *IL1B*, *IL32*, *IL36G*, *IL6* and *IL8*, were also upregulated in SARS-CoV-2-infected NHBE cells along with SOCS3, which has been shown to function as an IL-6 inhibitor [58,59]. This contradiction may be due to the imbalanced host response to SARS-CoV-2 as previously proposed [24]. For example, SARS-CoV-2 may interrupt the orchestration of a fine-tuned interplay between chemokines, interleukins and various types of recruited immune cells through unknown mechanisms. Similarly, a recent study [60] showed that the IAV infection not only induced IL-6 expression, but also upregulated SOCS3 in vitro and in vivo. The consequence of these gene expression changes needs to be investigated in the near future. Second, we found that SARS-CoV-2 infection in NHBE cells enhanced expression levels of some interesting gene sets that may explain molecular pathogenesis of COVID-19. For example, lung fibrosis-associated genes (*CSF3*, *IL8*, *CSF2*, *IL6*, *TNF*, *MMP9*, *IL1B* and *PDGFB*) were significantly upregulated in SARS-CoV-2-infected NHBE cells (Figure 2B). In addition, the genes associated with cornification, keratinization, epidermal cell differentiation, negative regulation of interferon gamma production, peptide cross linking, and protease inhibitor pathways were significantly upregulated (Figure 3, Figure 4 and Tables S3 and S4). These cornification (or keratinization)-associated genes are normally expressed by the stratified epithelia of the skin [61] and human airway basal cells [62], but SARS-CoV-2-infection appears to accelerate the process and ultimately lead to cell death by cornification [63]. Concordantly, the genes encoding transglutaminases (*TGM1*, *TGM2* and *TGM5*), which are enzymes responsible for crosslinking [64] and also involved in pulmonary fibrosis [65], were significantly upregulated. Our analysis further predicted that FOSL1 was one of the main transcription factors responsible for these processes, although additional and thorough examination is required. Lastly, elevated levels of the identified proinflammatory cytokines, such as TNF, IL-6 and IL-8, are associated with severe lung injury and adverse outcomes of SARS-CoV or MERS-CoV infections [7,12,13,14]. Similarly, severe COVID-19 patients have higher concentrations of IL-2, IL-6, IL-8 and TNF in the serum than mild cases, suggesting that the magnitude of cytokine storm is associated with the severity of the disease [66,67].

The molecular pathogenesis of COVID-19 inferred in this study implied that SARS-CoV-2-infected NHBE cells shared some features with those observed in COPD patients. For example, Araya et al. [68] reported increased epithelial immunostaining for involucrin (*IVL*), which is a marker of squamous metaplasia that is associated with airway obstruction in COPD. The squamous metaplasia of epithelial cells was also regarded as one of the main pathologic lung changes observed in COVID-19 patients [69]. Interestingly, the *IVL* gene was significantly upregulated in SARS-CoV-2-infected NHBE cells (Figure 3A). In addition, the *IL1B*, *IL1A*, *S100A8*, *RHCG*, *KRT6B* and *SPRR1A* genes, which were highly upregulated in the SARS-CoV-2-infected NHBE cells, were reported to be upregulated during a process of the squamous metaplasia in the same study [68]. Furthermore, Zhang et al. [70] reported that expression levels of the *IL6*, *IL8* and *MMP1* genes were increased in fibroblasts derived from lung tissues of COPD patients and the levels of the same were correlated with COPD stages. These genes were also significantly upregulated in SARS-CoV-2-infected NHBE cells. Interestingly, a recent study based on the human protein interactome also identified a similarity between COVID-19 and COPD [71]. Therefore, we hypothesized that the incidence of COPD in patients diagnosed with COVID-19 would be lower than expected due to treatments such as tiotropium. Strikingly, recent reports supported our hypothesis in that COPD was not listed as a comorbidity for any patient [12,72]. Based on our proposed molecular pathology of COVID-19, the lower reported prevalence of COPD or asthma in COVID-19 patients may be due to therapies used by patients.

Tiotropium—or similar treatments that the patients are receiving—would have several beneficial effects against SARS-CoV-2’s pathogenicity according to our proposed molecular pathogenesis of COVID-19. Tiotropium bromide has a favorable safety profile [18] and inhibits pulmonary neutrophilic inflammation in a concentration-dependent manner [73]. In addition, a recent study reported that inhaled corticosteroids (ICS), which are treatment options for COPD patients, downregulated the SARS-CoV-2 receptor ACE2 in COPD through suppression of type I interferon [13]. Nevertheless, this study has the following limitations. First, all the results from this study were derived by reanalyzing transcriptomic changes between SARS-CoV-2-infected and mock-infected NHBE cells. Therefore, the relationship between cytokine dysregulation in bronchial epithelial cells and cytokine storms observed in severe COVID-19 patients cannot be explained directly with these results. Second, this study is a descriptive research based on comprehensive molecular pathway analyses using in silico analysis methods. Therefore, the interplay between lung epithelial cells, endothelial cells, dendritic cells and macrophages, which are responsible for cytokine storms observed in severe COVID-19 patients, could not be addressed. Lastly, although tiotropium has been predicted to be a promising drug, according to the pathway analyses and the literature search in this study, tiotropium or therapies used in patients with COPD or asthma should be tested carefully in vitro and in vivo studies, as well as in clinical trials for COVID-19. In addition, the possibility that tiotropium may directly interact with the SARS-CoV-2’s replication complex, as predicted previously [22], should also be investigated.

We provide all the results (Tables S1–S4) used in this study for collective intelligence, so that researchers with various expertise can investigate them with different views and hope to discover more therapeutic options for those suffering from the COVID-19 pandemic.

## Figures and Tables

**Table 1:** Experimental evidence from the literature. A given drug can reduce (favored) or exacerbate (unfavored) the symptoms of COVID-19 by acting on the target(s).

Rank	Name	MT–DTI Affinity Score	Known Target or Phenotype (Effect)	Tissue or Cells (Species)	Reference
1	Rapamycin (Sirolimus)	8.835	IL-6 (decreased), IL-8 (decreased)	Pulmonary vascular endothelial cells and pulmonary-artery smooth muscle cells (human)	[44]
			SOCS3 (increased)	Th17 cells (mouse)	[47]
			NF-kB (decreased)	Lung tissue (mouse)	[43]
			Neutrophilic inflammation (decreased)		
			Lung injury (induced)		
			MERS-CoV (inhibited)	Hepatocyte-derived epithelial-like Huh7 cell (human)	[48]
2	Tiotropium Bromide	8.236	NFKB1 (decreased), RELA (decreased), ICAM1 (decreased)	Rhinovirus-infected airway epithelial cells (human)	[40]
			IL-6 (decreased), IL-8 (decreased), ICAM1 (decreased)	RSV-infected human epithelial type 2 cells (human)	[41]
			IL-6 (decreased), IL-1B (decreased), IRB-induced lung inflammation (decreased)	Inspiratory resistive breathing (IRB)-induced lung tissue (rat)	[49]
			IL-8 (decreased), proinflammation (decreased)	SV40 large T antigen-transformed 16HBE cells (human)	[50]
			MMP1 (decreased)	Lung fibroblasts, which were obtained from patients’ healthy tissue area, induced by transforming growth factor beta (human)	[51]
			IL-6 (decreased), TNF (decreased)	Lung tissue exposed to cigarette smoke and infected with RSV (mouse)	[42]
			IL-8 (decreased)	LPS-stimulated BEAS-2B cells and lung fibroblasts from patient’s healthy tissue area (human)	[52]
			TNF alpha-mediated chemotactic properties of stimulated alveolar macrophage (inhibited)	LPS-induced alveolar macrophage collected from COPD patients (human)	[53]
			IL-1B (decreased), TNF (decreased), interstitial fibrosis and inflammation (decreased)	Cigarette smoked-exposed lung tissue (mouse)	[54]
			IL-6 (decreased), IL-8 (decreased), TNF (decreased)	Cigarette smoked-exposed lung tissue (cat)	[55]

## Supplementary Materials

The following are available online at https://www.mdpi.com/1999-4915/12/7/776/s1, Table S1: Differentially expressed genes between groups; Table S2: Gene ontology analysis using g:Profiler with DEGs; Table S3: Gene set enrichment analysis using FGSEA; Table S4: PANTHER analysis.
